# Balancing right ventricular paced and right bundle branch activation to electrically optimize cardiac resynchronization therapy: triple-fusion pacing

**DOI:** 10.1093/europace/euae084

**Published:** 2024-04-23

**Authors:** Brian Wisnoskey, Niraj Varma

**Affiliations:** Cardiac Electrophysiology, Heart and Vascular Institute, Cleveland Clinic, 9500 Euclid Avenue, Cleveland, OH 44195, USA; Cardiac Electrophysiology, Heart and Vascular Institute, Cleveland Clinic, 9500 Euclid Avenue, Cleveland, OH 44195, USA

**Keywords:** Cardiac resynchronization therapy, LBBB, Electrical optimization, QRS narrowing, Device programming, Triple fusion

Cardiac resynchronization therapy (CRT) aims to restore electrical resynchronization. Success should be reflected by electrical measure(s).^1^ QRS optimization on an individualized basis is standard practice with conduction system pacing (CSP) but largely ignored with CRT (devices are left at nominal settings).^2^ A one-size-fits all solution may underlie disappointing results with device-based CRT algorithms (e.g. LV-fusion^3^). Therefore, we systematically evaluated QRS optimization using paced AV interval programming with CRT, contrasting biventricular (BiV: LV&RV) vs. LV-only fusion modes.

QRS duration (QRSd) narrowing usually is used to report resynchronization with CRT. An additional element is preservation of the rapid rS inscription in V_1_/V_2_ (‘rapid intrinsic’) generated by normal intrinsic right bundle branch (iRBB) conduction.^4,5^ Intrinsic right bundle branch conduction is essential to ‘LV fusion’ with LV-only pacing or ‘triple fusion’ with BiV pacing.^3,6,7^ Intrinsic right bundle branch contribution increases at longer paced atrioventricular delay (pAVD) relative to intrinsic AVD.

We tested effect of pAVD adjustment to achieve best electrical resynchronization in heart failure patients with Strauss-type LBBB (*n* = 40, age 68 ± 8 years; 22 (55%) male; 13 (35%) ischaemic cardiomyopathy; LVEF 25 ± 8%). Native intervals were PR 206 ± 35 ms; rS 69 ± 12 ms and QRSd 171 ± 15 ms. The qLV/QRS was 73 ± 9%, indicating optimized LV lead position. RV-only pacing without fusion prolonged QRSd (210 ± 23 ms) and rS (104 ± 17 ms) intervals.

Paced QRSd and rS (r wave onset to S wave nadir in V_1_/V_2_) were measured using simultaneous 12-lead recordings during nominal BiV (BiV_Nom_; pAVD 140/110 ms) and then during each programmed setting. In each patient, pAVD was adjusted (in increments of ∼5%) from 60% to 95% of the PR interval, firstly during BiV (simultaneous LV&RV) and then LV-only pacing. Optimal AVDs (BiV_Opt_ and LV-only_Opt_) were determined by the narrowest QRSd and then rS measured (representative ECG presented in *Figure 1A*).

**Figure 1:**
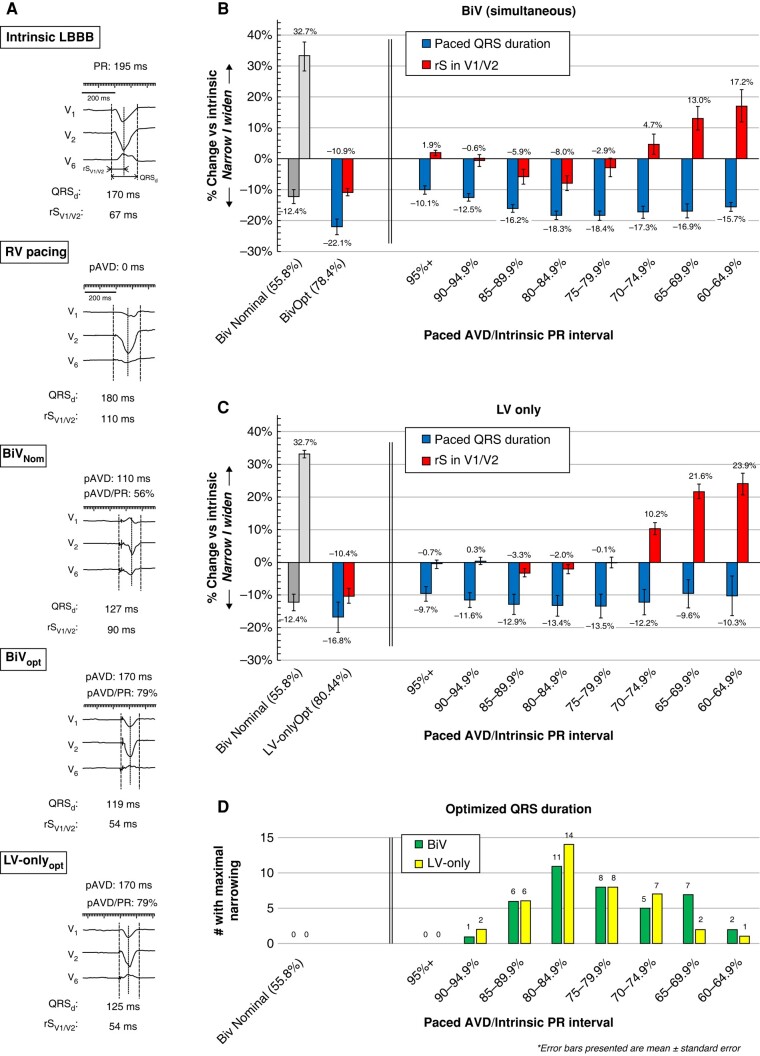
Electrical optimization as a function of pAVD/PR interval programming.

*Figure 1B* depicts summary data for QRS and rS intervals during BiV pacing. BiV_Nom_ narrowed QRSd by 12.4% (darker grey) but widened rS interval (lighter grey) vs. intrinsic LBBB. Progressive pAVD/PR lengthening abbreviated QRSd and rS. Maximum effects were seen in range 75–85%. BiV_Opt_ reduced QRSd by 22.1% vs. intrinsic LBBB and was associated with longer pAVD than BiV_Nom_ [78 ± 7 vs. 56 ± 7% of PR interval (absolute intervals 106–276 ms)]. When compared, BiV_Nom_ produced inferior resynchronization: 12.3% wider QRS than BiV_opt_ (149 ± 17 vs. 133 ± 15 ms) and 48% wider rS (89 ± 20 vs. 60 ± 9 ms); all *P* < 0.05 (maximal QRS narrowing is calculated as the mean from each individual patient whereas data presented in the figures is aggregated for each pAVD/PR interval).

LV-only pacing (*Figure 1C*) showed maximum QRSd shortening at pAVD/PR range 75–85% (absolute intervals 115–297 ms) and rS abbreviation at 85–90% (a broadly similar range to BiV). LV-only_Opt_ reduced QRSd duration by 16.8% vs. intrinsic LBBB, marginally superior to BiV_Nom_ (142 ± 16 ms vs. 149 ± 17 ms; *P* < 0.05), but inferior to BiV fusion pacing (BiV_Opt_ 133 ± 15; *P* < 0.05 ms). Individually, LV-only_Opt_ programming was superior to BiV_Opt_ infrequently, achieving narrowest QRS in only 4/40 patients (10%).

Notably, max QRS shortening with both BiV_Opt_ and LV-only_Opt_ was associated with minimized rS interval suggesting that iRBB contributes to electrical resynchronization in both fusion modes. pAVD/PR range 80–85% was optimal in most patients (*Figure 1D*) but notably in some was <65% or >90%.

Important points for CRT programming are highlighted: (1) nominal settings usually underreach maximal optimization; (2) individualized adjustment of the paced AVD is key as optimal values are widely distributed; (3) electrical resynchronization is best achieved via incorporation of rapid iRBB conduction (with either LV or BiV fusion pacing); (4) shortening pAVD truncates iRBB contribution (broadening rS) and diminishes magnitude of QRS narrowing; and (5) resynchronization is significantly enhanced by the incorporation of RV pacing to LV and iRBB (i.e. triple fusion) but requires longer pAVD/PR intervals.

The incorporation of the RV paced wavefront to *improve* electrical resynchronization, as demonstrated here, may be counterintuitive. Although RV pacing is deleterious when ventricular activation in HF patients is *fully* committed to this wavefront, its *initial* effects on septal activation to break down functional conduction barriers may synergize with iRBB conduction and facilitate resynchronization with LV pacing.^8,9^ pAVD timing is crucial to titrate this effect.^10^

Our findings may explain the neutral results of trials testing CRT device algorithms that apply a one-size-fits all solution.^3,11^ For example, the LV-only fusion pacing algorithm uses a non-programmable paced pAVD of ∼70% of the PR interval. We show optimal pAVD for this mode is widely distributed and more likely to be longer (>80% of the PR) that enables greater iRBB contribution. However, in 90% of patients, this was inferior to BiV_Opt_. Thus, adding RV pacing at longer pAVD (triple fusion) further enhances electrical resynchronization.^7^ Notably, individualized CRT programming as demonstrated here results in similar QRSd to those reported with CSP.^12–14^

In conclusion, individualized programming to balance iRBB, RV, and LV paced wavefronts is key to electrical resynchronization and may improve CRT outcomes. This hypothesis is being tested in the SyncAV (NCT04100148) randomized trial.

**Conflict of interest:** B.W.: employee of Abbott. N.V.: Abbott, Biotronik, Boston Scientific, EP Solutions, Implicity, Impulse Dynamics, Medtronic, Pacemate.

## Data Availability

Raw data will be available on reasonable request to the corresponding author.
